# 2,6-Di­amino-4-chloro­pyrimidine–succinic acid (2/1)

**DOI:** 10.1107/S2414314620012390

**Published:** 2020-09-15

**Authors:** Nandhini Chakkarapani, Suganya Murugan, Abdul Razak Ibrahim, Savaridasan Jose Kavitha, Madhukar Hemamalini, Venkatachalam Rajakannan

**Affiliations:** a Department of Crystallography and Biophysics, University of Madras, Guindy Campus, Chennai-600 025, Tamil Nadu, India; b Department of Chemistry, Mother Teresa Women’s University, Kodaikanal, Tamil Nadu, India; c School of Physics, Universiti Sains Malaysia, 11800 USM, Penang, Malaysia; University of Aberdeen, Scotland

**Keywords:** crystal structure, co-crystal, Hirshfeld surface analysis.

## Abstract

In the title 2:1 co-crystal, 2C_4_H_5_ClN_4_·C_4_H_6_O_4_ the complete succinic acid mol­ecule is generated by a crystallographic centre of symmetry. In the crystal, pairwise O—H⋯N and N—H⋯O hydrogen bonds link the pyrimidine and succinic acid mol­ecules, generating *R*_2_^2^(8) loops. The pyrimidine mol­ecules are linked by pairwise N—H⋯N hydrogen bonds, again generating *R*_2_^2^(8) loops. Collectively, the hydrogen bonds link the components into corrugated (100) sheets. The Hirshfeld surface is presented.

## Abstract 2

In the title 2:1 co-crystal, the complete succinic acid mol­ecule is generated by a crystallographic centre of symmetry.

## Graphical abstract

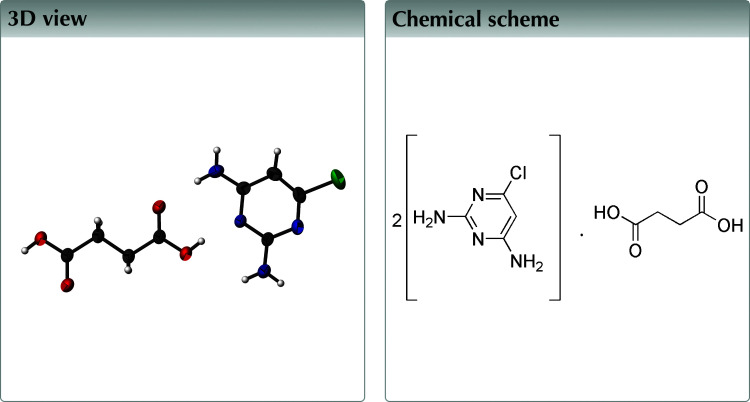

## Structure description

Some amino­pyrimidine derivatives are used as anti­folate drugs (Hunt *et al.*, 1980 ▸). The crystal structures of various amino­pyrimidine derivatives (Schwalbe & Williams, 1982 ▸; Edison *et al.*, 2014 ▸; Thanigaimani *et al.*, 2012 ▸) have been reported. In the present study, the synthesis and structure of the title 2:1 co-crystal are described.

**Figure 1:**
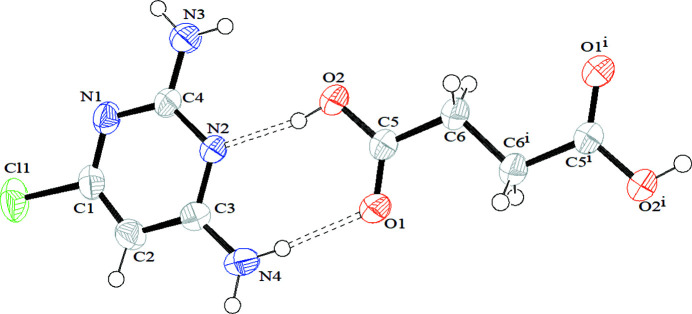
The mol­ecular structure of the title compound showing 50% displacement ellipsoids and hydrogen bonds indicated by dashed lines. Symmetry code: (i) 2 − *x*, 3 − *y*, 1 − *z*.

The complete succinic acid mol­ecule is generated by a crystallographic centre of symmetry (Fig. 1 ▸), with key torsion angles O1—C5—C6—C6^i^ = −2.5 (3)° and O2—C5—C6—C6^i^ = 178.28 (18)° [symmetry code: (i) 2 − *x*, 3 − *y*, 1 − *z*].

**Figure 2:**
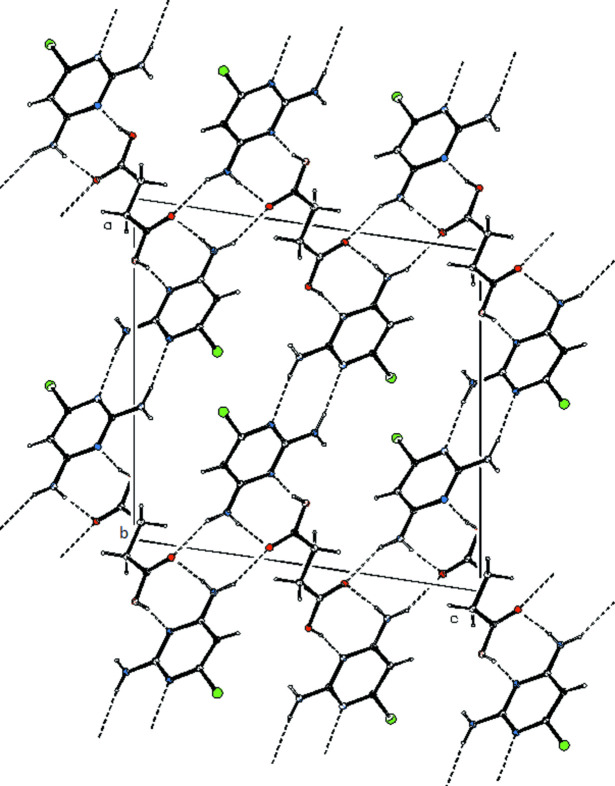
Partial packing diagram of the title compound showing hydrogen bonds as dashed lines.

In the crystal, pairwise O2—H2⋯N2 and N4—H4*A*··O1 hydrogen bonds (for symmetry codes, see Table 1 ▸) link the pyrimidine and succinic acid mol­ecules, generating 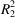 (8) loops. The mean planes of the succinic acid and linked pyrimidine mol­ecules are close to parallel [dihedral angle = 8.67 (6)°]. The pyrimidine mol­ecules are linked by pairwise N3—H3*A*⋯N1^i^ hydrogen bonds, again generating 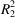 (8) loops. An N4—H4*B*⋯O1^ii^ hydrogen bond also links the pyrimidine and succinic acid species. Collectively, the hydrogen bonds link the components into corrugated (100) sheets (Fig. 2 ▸).

**Figure 3:**
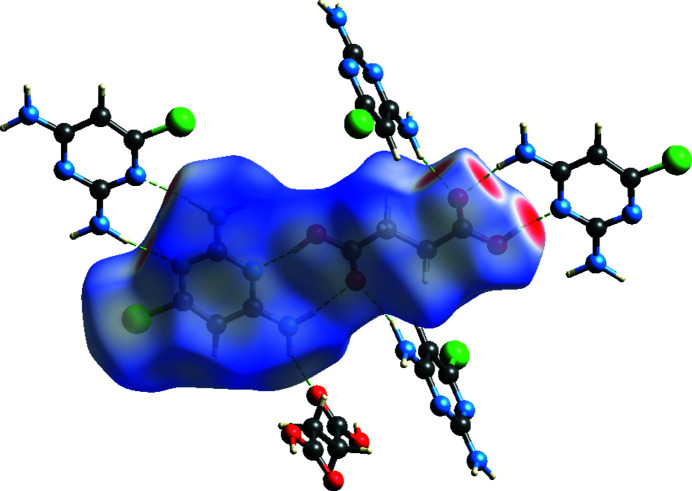
The Hirshfeld surface mapped over *d*_norm_ for the title compound.

The Hirshfeld surface (Turner *et al.*, 2017 ▸) of the pyrimidine–succinic acid grouping is shown in Fig. 3 ▸, where red spots represent short inter­molecular contacts associated with the various hydrogen bonds. The most significant contact percentages arising from two-dimensional fingerprint plots are: H⋯H = 32.5%, O⋯H/H⋯O = 19.7%, N⋯H/H⋯N = 13.6%, Cl⋯H/H⋯Cl = 7.9%, H⋯C/C⋯H = 5.5% and O⋯C/C⋯O = 4.8%. Other contact types contribute a negligible amount.

## Synthesis and crystallization

A 10 ml methano­lic solution (hot) of 2,6-di­amino-4-chloro­pyrimidine (32 mg) and a 10 ml aqueous solution (hot) of succinic acid (29 mg) were mixed and heated for 10 min and then cooled to room temperature. Colourless blocks grew over the course of a few days as the solvents evaporated.

## Refinement

Crystal data, data collection and structure refinement details are summarized in Table 2 ▸.

## Supplementary Material

Crystal structure: contains datablock(s) global, I. DOI: 10.1107/S2414314620012390/hb4365sup1.cif

Structure factors: contains datablock(s) I. DOI: 10.1107/S2414314620012390/hb4365Isup2.hkl

Supporting information file. DOI: 10.1107/S2414314620012390/hb4365Isup3.cml

CCDC reference: 2003667

Additional supporting information:  crystallographic information; 3D view; checkCIF report

## Figures and Tables

**Table 1:** Hydrogen-bond geometry (Å, °)

*D*—H⋯*A*	*D*—H	H⋯*A*	*D*⋯*A*	*D*—H⋯*A*
O2—H2⋯N2	0.82	1.81	2.622 (2)	169
N3—H3*A*⋯N1^i^	0.86	2.19	3.048 (2)	173
N4—H4*A*⋯O1	0.86	1.94	2.794 (2)	169
N4—H4*B*⋯O1^ii^	0.86	2.04	2.8510 (19)	156

Symmetry codes: (i) 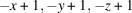 ; (ii) 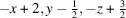 .

**Table 2:** Experimental details

Crystal data	
Chemical formula	2C_4_H_5_ClN_4_·C_4_H_6_O_4_
*M*_r_	407.23
Crystal system, space group	Monoclinic, *P*2_1_/*c*
Temperature (K)	296
*a*, *b*, *c* (Å)	13.2096 (14), 4.9765 (5), 13.5673 (14)
β (°)	98.603 (2)
*V* (Å^3^)	881.85 (16)
*Z*	2
Radiation type	Mo *K*α
μ (mm^−1^)	0.41
Crystal size (mm)	0.72 × 0.34 × 0.13

Data collection	
Diffractometer	Bruker SMART APEXII CCD
Absorption correction	Multi-scan (*SADABS*; Krause *et al.*, 2015 ▸)
*T*_min_, *T*_max_	0.631, 0.746
No. of measured, independent and observed [*I* > 2σ(*I*)] reflections	15031, 2599, 2102
*R*_int_	0.032
(sin θ/λ)_max_ (Å^−1^)	0.707

Refinement	
*R*[*F* ^2^ > 2σ(*F* ^2^)], *wR*(*F* ^2^), *S*	0.043, 0.128, 1.04
No. of reflections	2599
No. of parameters	118
H-atom treatment	H-atom parameters constrained
Δρ_max_, Δρ_min_ (e Å^−3^)	0.33, −0.44

Computer programs: *APEX2*, *SAINT* and *XPREP* (Bruker, 2009 ▸), *SHELXT* (Sheldrick, 2015*a* ▸), *SHELXL2018/3* (Sheldrick, 2015*b* ▸) and *PLATON* (Spek, 2020 ▸).

## full crystallographic data

### Crystal data

**Table:**

2C_4_H_5_ClN_4_·C_4_H_6_O_4_	*F*(000) = 420
*M**_r_* = 407.23	*D*_x_ = 1.534 Mg m^−^^3^
Monoclinic, *P*2_1_/*c*	Mo *K*α radiation, λ = 0.71073 Å
*a* = 13.2096 (14) Å	Cell parameters from 3778 reflections
*b* = 4.9765 (5) Å	θ = 2.6–29.9°
*c* = 13.5673 (14) Å	µ = 0.41 mm^−^^1^
β = 98.603 (2)°	*T* = 296 K
*V* = 881.85 (16) Å^3^	Plate, colourless
*Z* = 2	0.72 × 0.34 × 0.13 mm

### Data collection

**Table:**

Bruker SMART APEXII CCD diffractometer	2102 reflections with *I* > 2σ(*I*)
Radiation source: fine-focus sealed tube	*R*_int_ = 0.032
ω and φ scan	θ_max_ = 30.2°, θ_min_ = 1.6°
Absorption correction: multi-scan (SADABS; Krause *et al.*, 2015)	*h* = −18→18
*T*_min_ = 0.631, *T*_max_ = 0.746	*k* = −7→7
15031 measured reflections	*l* = −18→19
2599 independent reflections	

### Refinement

**Table:**

Refinement on *F*^2^	Primary atom site location: structure-invariant direct methods
Least-squares matrix: full	Secondary atom site location: difference Fourier map
*R*[*F*^2^ > 2σ(*F*^2^)] = 0.043	Hydrogen site location: inferred from neighbouring sites
*wR*(*F*^2^) = 0.128	H-atom parameters constrained
*S* = 1.04	*w* = 1/[σ^2^(*F*_o_^2^) + (0.0642*P*)^2^ + 0.3195*P*] where *P* = (*F*_o_^2^ + 2*F*_c_^2^)/3
2599 reflections	(Δ/σ)_max_ = 0.001
118 parameters	Δρ_max_ = 0.33 e Å^−^^3^
0 restraints	Δρ_min_ = −0.44 e Å^−^^3^

### Special details

**Table:**

Geometry. All esds (except the esd in the dihedral angle between two l.s. planes) are estimated using the full covariance matrix. The cell esds are taken into account individually in the estimation of esds in distances, angles and torsion angles; correlations between esds in cell parameters are only used when they are defined by crystal symmetry. An approximate (isotropic) treatment of cell esds is used for estimating esds involving l.s. planes.
Refinement. The hydrogen atoms were positioned geometrically (C—H = 0.93–0.97 Å, N—H = 0.86) Å, O—H =0.82 Å) and were refined using a riding model with *U*_iso_(H) = 1.2 *U*_eq_(carrier).

### Fractional atomic coordinates and isotropic or equivalent isotropic displacement parameters (Å^2^)

**Table:**

	*x*	*y*	*z*	*U*_iso_*/*U*_eq_	
Cl1	0.58724 (4)	0.10526 (9)	0.74117 (4)	0.06071 (19)	
N2	0.74496 (9)	0.7549 (3)	0.60431 (9)	0.0345 (3)	
O2	0.81782 (9)	1.1416 (3)	0.50401 (9)	0.0504 (3)	
H2	0.803150	1.017333	0.538975	0.076*	
O1	0.96386 (9)	1.1222 (3)	0.60813 (9)	0.0523 (3)	
N1	0.60385 (10)	0.4544 (3)	0.60332 (10)	0.0400 (3)	
C5	0.90949 (11)	1.2237 (3)	0.53630 (11)	0.0367 (3)	
C2	0.74276 (13)	0.4470 (3)	0.73964 (12)	0.0420 (4)	
H2A	0.771513	0.374017	0.800544	0.050*	
C4	0.65260 (11)	0.6548 (3)	0.56340 (11)	0.0358 (3)	
C6	0.94673 (11)	1.4522 (3)	0.47896 (12)	0.0391 (3)	
H6A	0.899454	1.601474	0.478469	0.047*	
H6B	0.946393	1.395885	0.410457	0.047*	
N4	0.88038 (11)	0.7585 (3)	0.73071 (11)	0.0514 (4)	
H4A	0.906878	0.884453	0.699449	0.062*	
H4B	0.912066	0.700416	0.786646	0.062*	
C1	0.65147 (12)	0.3614 (3)	0.68959 (12)	0.0392 (3)	
C3	0.79074 (11)	0.6540 (3)	0.69271 (11)	0.0364 (3)	
N3	0.60778 (11)	0.7630 (3)	0.47853 (11)	0.0532 (4)	
H3A	0.549166	0.704581	0.450636	0.064*	
H3B	0.637362	0.891492	0.451444	0.064*	

### Atomic displacement parameters (Å^2^)

**Table:**

	*U*^11^	*U*^22^	*U*^33^	*U*^12^	*U*^13^	*U*^23^
Cl1	0.0568 (3)	0.0454 (3)	0.0867 (4)	0.00090 (19)	0.0328 (3)	0.0224 (2)
N2	0.0290 (6)	0.0381 (6)	0.0350 (6)	−0.0071 (5)	0.0006 (4)	0.0029 (5)
O2	0.0348 (6)	0.0595 (8)	0.0529 (7)	−0.0178 (5)	−0.0068 (5)	0.0173 (6)
O1	0.0417 (6)	0.0602 (8)	0.0495 (7)	−0.0200 (6)	−0.0111 (5)	0.0144 (6)
N1	0.0340 (6)	0.0392 (7)	0.0473 (7)	−0.0080 (5)	0.0078 (5)	0.0021 (5)
C5	0.0322 (7)	0.0381 (7)	0.0390 (7)	−0.0070 (6)	0.0028 (5)	−0.0001 (6)
C2	0.0424 (8)	0.0426 (8)	0.0415 (8)	0.0049 (6)	0.0077 (6)	0.0102 (6)
C4	0.0304 (6)	0.0397 (7)	0.0366 (7)	−0.0074 (6)	0.0028 (5)	−0.0005 (6)
C6	0.0324 (7)	0.0383 (8)	0.0456 (8)	−0.0076 (6)	0.0026 (6)	0.0048 (6)
N4	0.0412 (7)	0.0614 (10)	0.0462 (8)	−0.0086 (7)	−0.0113 (6)	0.0121 (7)
C1	0.0379 (7)	0.0333 (7)	0.0495 (8)	0.0011 (6)	0.0170 (6)	0.0052 (6)
C3	0.0336 (7)	0.0379 (7)	0.0372 (7)	0.0017 (6)	0.0030 (5)	0.0007 (6)
N3	0.0432 (7)	0.0651 (10)	0.0460 (8)	−0.0230 (7)	−0.0102 (6)	0.0136 (7)

### Geometric parameters (Å, º)

**Table:**

Cl1—C1	1.7356 (16)	C2—H2A	0.9300
N2—C3	1.3561 (18)	C4—N3	1.327 (2)
N2—C4	1.3572 (18)	C6—C6^i^	1.514 (3)
O2—C5	1.2911 (17)	C6—H6A	0.9700
O2—H2	0.8200	C6—H6B	0.9700
O1—C5	1.2294 (19)	N4—C3	1.325 (2)
N1—C1	1.327 (2)	N4—H4A	0.8600
N1—C4	1.3443 (19)	N4—H4B	0.8600
C5—C6	1.501 (2)	N3—H3A	0.8600
C2—C1	1.361 (2)	N3—H3B	0.8600
C2—C3	1.410 (2)		
			
C3—N2—C4	118.71 (13)	C5—C6—H6B	108.8
C5—O2—H2	109.5	C6^i^—C6—H6B	108.8
C1—N1—C4	114.95 (13)	H6A—C6—H6B	107.7
O1—C5—O2	123.08 (14)	C3—N4—H4A	120.0
O1—C5—C6	121.62 (13)	C3—N4—H4B	120.0
O2—C5—C6	115.30 (13)	H4A—N4—H4B	120.0
C1—C2—C3	115.32 (14)	N1—C1—C2	126.81 (14)
C1—C2—H2A	122.3	N1—C1—Cl1	114.59 (12)
C3—C2—H2A	122.3	C2—C1—Cl1	118.61 (13)
N3—C4—N1	118.16 (13)	N4—C3—N2	116.94 (14)
N3—C4—N2	117.58 (14)	N4—C3—C2	123.14 (14)
N1—C4—N2	124.26 (14)	N2—C3—C2	119.91 (14)
C5—C6—C6^i^	113.62 (16)	C4—N3—H3A	120.0
C5—C6—H6A	108.8	C4—N3—H3B	120.0
C6^i^—C6—H6A	108.8	H3A—N3—H3B	120.0
			
C1—N1—C4—N3	−178.01 (15)	C4—N1—C1—Cl1	179.33 (11)
C1—N1—C4—N2	1.9 (2)	C3—C2—C1—N1	−0.8 (3)
C3—N2—C4—N3	177.81 (15)	C3—C2—C1—Cl1	179.50 (12)
C3—N2—C4—N1	−2.1 (2)	C4—N2—C3—N4	−179.40 (14)
O1—C5—C6—C6^i^	−2.5 (3)	C4—N2—C3—C2	0.7 (2)
O2—C5—C6—C6^i^	178.28 (18)	C1—C2—C3—N4	−179.27 (16)
C4—N1—C1—C2	−0.4 (2)	C1—C2—C3—N2	0.6 (2)

Symmetry code: (i) −*x*+2, −*y*+3, −*z*+1.

### Hydrogen-bond geometry (Å, º)

**Table:**

*D*—H···*A*	*D*—H	H···*A*	*D*···*A*	*D*—H···*A*
O2—H2···N2	0.82	1.81	2.622 (2)	169
N3—H3*A*···N1^ii^	0.86	2.19	3.048 (2)	173
N4—H4*A*···O1	0.86	1.94	2.794 (2)	169
N4—H4*B*···O1^iii^	0.86	2.04	2.8510 (19)	156

Symmetry codes: (ii) −*x*+1, −*y*+1, −*z*+1; (iii) −*x*+2, *y*−1/2, −*z*+3/2.
